# Association of Circulating Irisin with Adiposity, Sarcopenia-Related Indices, and Phenotypes in Premenopausal and Postmenopausal Women

**DOI:** 10.3390/nu18213531

**Published:** 2026-10-27

**Authors:** Nouf Aljawini, Syed Shahid Habib, Khalid Al-Regaiey

**Affiliations:** 1 Department of Community Health Sciences, College of Applied Medical Sciences, King Saud University, Riyadh 11433, Saudi Arabia; 2 Department of Physiology, College of Medicine, King Saud University, Riyadh 11416, Saudi Arabia

**Keywords:** irisin, adipo-myokine, obesity, sarcopenia, sarcopenic obesity, menopause, adiposity, muscle strength

## Abstract

**Background:** Irisin is an exercise-induced myokine also produced by adipose tissue and thus considered an adipo-myokine. Its relationship with adiposity and sarcopenia-related indices remains unclear. We examined these associations and compared circulating irisin concentrations across operationally defined phenotypes in premenopausal and postmenopausal women. **Methods:** In this cross-sectional study, 79 women underwent body-composition assessment by BIA and HGS measurement by dynamometry, of whom 78 underwent serum irisin measurement by ELISA. Participants were classified into four operational phenotypes (Control, Obesity, Sarcopenia, and Sarcopenic Obesity) using BMI ≥ 30 kg/m^2^ for obesity and HGS < 16 kg for low muscle strength. Irisin was compared across phenotypes and correlated with adiposity and sarcopenia-related indices. **Results:** Circulating irisin was significantly lower in women with BMI-defined obesity than in those without obesity (173.70 [89.03–357.20] vs. 347.30 [188.90–716.00] pg/mL; *p* = 0.0024) but did not differ when obesity was defined by %BF (≥35%; *p* = 0.1034). Overall, irisin did not differ by menopausal status. Among postmenopausal women, irisin concentrations were lower in those with obesity than in those without obesity (179.70 [85.30–321.90] vs. 403.60 [219.20–801.70] pg/mL; *p* = 0.0043), whereas no significant difference was observed among premenopausal women (*p* = 0.2666). Across the operational phenotypes, irisin was lowest in the sarcopenic obesity group (128.90 [68.57–321.90] pg/mL) and significantly lower than in controls (322.40 [164.10–498.40] pg/mL; *p* = 0.0076). Irisin was not significantly associated with HGS, ASM, or MQI, whereas ASMI showed an inverse association (r = −0.2773, *p* = 0.0153). Significant inverse associations were observed with FMI (r = −0.3835, *p* = 0.0006), BMI (r = −0.3609, *p* = 0.0013), and %BF (r = −0.3396, *p* = 0.0025). In exploratory univariable regression analyses, BMI, FMI, and %BF accounted for 15.9%, 15.2%, and 8.4% of the variance in circulating irisin, respectively. **Conclusions:** Circulating irisin showed consistent inverse associations with adiposity indices but a less consistent pattern across muscle-related indices, suggesting that adiposity may be particularly relevant to circulating irisin in women.

## 1. Introduction

Sarcopenia is characterized by progressive impairment of skeletal muscle mass and function and is associated with falls, fractures, disability, and mortality [1]. Rosenberg introduced the term in 1989 after observing differences in thigh lean mass between an older and a younger woman [2]. Since then, the concept has evolved from an emphasis on muscle mass toward a broader construct incorporating muscle strength and physical performance, reflected in definitions developed by international groups and societies [3,4,5,6,7,8]. The European Working Group on Sarcopenia in Older People (EWGSOP2) now prioritizes reduced muscle strength for sarcopenia detection, with reduced muscle quantity or quality supporting confirmation and impaired physical performance defining severe sarcopenia [1,4]. This shift reflects the recognition that muscle mass and strength do not necessarily decline in parallel. Handgrip strength (HGS) is a practical measure of muscle strength, associated with strength in other muscle groups and predictive of adverse outcomes including functional impairment, prolonged hospitalization, reduced quality of life, and mortality [1,9,10]. Accordingly, HGS is widely recommended for assessing muscle strength in clinical and community settings [1].

The coexistence of excess adiposity and impaired muscle function defines sarcopenic obesity, a distinct phenotype with potentially greater metabolic and functional consequences than either condition alone [11]. First described by Heber and colleagues as reduced lean mass accompanied by excess body fat [12], sarcopenic obesity represents the convergence of altered adiposity and skeletal muscle function. Obesity may further compromise skeletal muscle through metabolic and tissue-level mechanisms, including fat infiltration, thereby compounding the consequences of impaired muscle function [11]. Identifying biological factors that reflect the interaction between adipose tissue and skeletal muscle may therefore provide insight into this phenotype.

Myokines have established skeletal muscle as an endocrine organ capable of communicating with other tissues through autocrine, paracrine, and endocrine signaling [13]. Irisin is an exercise-induced myokine generated through cleavage of fibronectin type III domain-containing protein 5 and is also produced by adipose tissue, supporting its characterization as an adipo-myokine [14]. Exercise-induced irisin has been associated with increased uncoupling protein 1 expression and adipose tissue browning, greater energy expenditure, improved glucose tolerance, and modest weight reduction [15]. Its association with both skeletal muscle and adipose tissue makes irisin particularly relevant to phenotypes in which impaired muscle function and excess adiposity coexist.

However, the relationship between circulating irisin and these phenotypes remains unresolved. Studies have reported inconsistent associations between irisin and skeletal muscle [16,17,18,19,20,21], while circulating irisin has been associated with muscle mass, BMI, glucose, age, insulin, and cholesterol, indicating complex relationships with metabolic status [22]. Irisin expression in adipose tissue has also been reported to be inversely associated with obesity and positively associated with markers of brown adipose tissue [23]. Irisin has consequently been proposed as a potential biomarker of sarcopenia [24], and Park et al. reported lower circulating irisin in women with sarcopenia than in women without sarcopenia [18]. Collectively, these findings implicate irisin in both adiposity- and muscle-related phenotypes, but do not establish whether circulating irisin is more closely associated with adiposity or impaired muscle strength, particularly when these features coexist.

Menopause provides an important context for examining this relationship because hormonal changes are accompanied by alterations in adiposity and skeletal muscle [25]. In Saudi women, low muscle strength has been observed despite relatively high muscle mass indices, highlighting the potential dissociation between muscle quantity and strength [26]. Postmenopausal women have also shown lower muscle strength than premenopausal women despite no significant differences in muscle mass indices [26], while Saudi postmenopausal women have higher adiposity indices and lower muscle mass indices than premenopausal women [27], consistent with findings across different racial and ethnic populations [28]. These changes are particularly relevant given the increasing burden of obesity among Saudi women [27,29,30] and suggest that menopausal status provides an important context for examining the relationship between irisin, adiposity, and muscle function.

Despite growing interest in irisin as an adipo-myokine, it remains unclear how circulating irisin relates to adiposity, muscle-strength impairment, and their coexistence as sarcopenic obesity. These relationships also remain insufficiently characterized across menopausal status. Therefore, we investigated the associations of circulating irisin with adiposity, muscle-strength impairment, and their coexistence as sarcopenic obesity in women, and explored these relationships within premenopausal and postmenopausal subgroups. We further assessed which adiposity and sarcopenia indices were most closely associated with circulating irisin. We hypothesized that obesity and its coexistence with sarcopenia would be associated with lower circulating irisin concentrations.

## 2. Materials and Methods

### 2.1. Study Design and Setting

This cross-sectional observational study was conducted at King Saud University Medical City (KSUMC), Riyadh, Saudi Arabia, between March 2022 and November 2023. Women were recruited consecutively during designated recruitment sessions from the Obstetrics and Gynecology and Family Medicine outpatient clinics. Eligibility was determined through clinical screening and review of medical records according to predefined criteria. Exclusion criteria included pregnancy, acute medical conditions, physical limitations precluding bioelectrical impedance analysis (BIA) or handgrip strength (HGS) assessment, and medical conditions or treatments likely to substantially affect body composition.

Of 82 women screened, three were excluded because injuries precluded BIA (two with palm injuries and one with a foot injury). Eligible women who provided written informed consent underwent standardized clinical evaluation, anthropometric and body-composition assessments, HGS testing, and fasting blood sampling. The final study population comprised 79 women, of whom 78 had circulating irisin measurements available for analysis. All 79 participants were also included in a previously published study involving 134 women [26]. Participant flow chart is illustrated in Supplementary Figure S1. All procedures were performed by trained healthcare professionals following standardized protocols. The study was reported in accordance with the Strengthening the Reporting of Observational Studies in Epidemiology (STROBE) guidelines [31].

### 2.2. Ethical Approval

The study was approved by the Institutional Review Board of the College of Medicine, King Saud University (No. E-21-5998) and conducted in accordance with the Declaration of Helsinki [32]. Written informed consent was obtained from all participants before study procedures were initiated.

### 2.3. Study Protocols and Procedures

#### 2.3.1. Medical and Menstrual History

Medical diagnoses, medication use and menopausal status were verified from medical records and participant interviews. Menstrual history was obtained using a standardized semi-structured interview. Menopausal status was classified according to the Stages of Reproductive Aging Workshop (STRAW) criteria [33]. Women were classified as premenopausal if they reported ongoing regular menstruation and as postmenopausal if they had experienced amenorrhea for ≥12 consecutive months.

#### 2.3.2. Clinical Evaluation

Resting blood pressure and pulse rate were measured using an automated vital-signs monitor (5300P, Welch Allyn, Skaneateles Falls, NY, USA). Participants rested seated for 10 min before two consecutive blood-pressure measurements were obtained. The mean SBP and DBP values were used for analysis. Mean arterial pressure (MAP) was calculated as MAP = [(2/3 × DBP) + (1/3 × SBP)]. Hypertension was defined as SBP ≥ 140 mmHg, DBP ≥ 90 mmHg, or current antihypertensive treatment [34].

Body weight and height were measured barefoot and in light clothing using a calibrated digital scale and stadiometer (Seca 576; SECA Corp., Hamburg, Germany), with participants positioned with the head in the Frankfort horizontal plane. Each measurement was obtained twice to the nearest 0.1 kg and 0.1 cm, respectively, and the mean values were used. BMI was calculated as weight (kg)/height (m^2^) and categorized according to established adult BMI criteria [35].

#### 2.3.3. Circulating Irisin Measurement

Circulating serum irisin concentrations were quantified using a commercially available enzyme-linked immunosorbent assay (ELISA) kit (abx251138; Abbexa Ltd., Cambridge, UK) according to the manufacturer’s instructions. Fasting morning blood (20 mL) was collected from the cubital vein into serum-separation tubes, allowed to clot at room temperature for 20 min, and centrifuged at 3000 rpm (1500× *g*) for 20 min at 4 °C. The separated serum was divided into aliquots and stored at −80 °C until analysis. All samples were analyzed in the same assay batch, in duplicate, by laboratory personnel blinded to phenotype and menopausal status. The mean concentration of duplicate measurements was used for subsequent analyses. The assay sensitivity was 9.38 pg/mL, with manufacturer-reported intra- and inter-assay coefficients of variation of <10%. Absorbance was measured at 450 nm using a Synergy HT microplate reader (Agilent, Santa Clara, CA, USA), and irisin concentrations were calculated from the assay-specific standard curve according to the manufacturer’s recommended procedure.

#### 2.3.4. Laboratory Measurements

Following an overnight fast of 8–10 h, venous blood samples were collected by a trained phlebotomist and processed within 2 h. Samples were centrifuged at 3000 rpm for 15 min, after which serum concentrations of total cholesterol (TC), high-density lipoprotein cholesterol (HDL-C), and triglycerides (TG) were quantified using a Cobas automated analyzer (Roche Diagnostics, Indianapolis, IN, USA). Low-density lipoprotein cholesterol (LDL-C) was calculated using the Friedewald equation [36], with lipid concentrations expressed in mmol/L: LDL-C = TC − HDL-C − TG/2.2.

#### 2.3.5. Body Composition Assessment

Body composition was assessed using a segmental, multifrequency bioelectrical impedance analyzer (MC-980; Tanita Corp., Tokyo, Japan). Measurements were obtained in the standing position using an eight-electrode configuration across the upper and lower limbs and trunk. The device provided estimates of FM, FFM and BF% using its proprietary algorithm, with segmental measurements used to derive appendicular skeletal muscle mass. FMI and FFMI were calculated as FM/height^2^ and FFM/height^2^, respectively. ASMI was calculated as appendicular skeletal muscle mass divided by height squared. BIA has previously demonstrated acceptable validity and reproducibility for estimating body composition relative to reference methods [37,38].

#### 2.3.6. Muscle Strength Assessment

Handgrip strength (HGS) was assessed using a digital hand dynamometer (T.K.K. 5401; Takei Scientific Instruments Co., Ltd., Niigata, Japan) according to the American Society of Hand Therapists protocol [39]. Participants were seated with the feet flat on the floor, the arm positioned alongside the trunk, the elbow flexed at 90°, and the wrist in a neutral position. Three maximal contractions were performed with the dominant hand, separated by 1 min intervals. The highest recorded value was used for analysis. The protocol has demonstrated high test–retest reliability (ICC = 0.99) [40].

### 2.4. Study Definitions and Outcomes

#### 2.4.1. Definition of Obesity, Sarcopenia and Sarcopenic Obesity

Obesity was defined as BMI ≥ 30 kg/m^2^ according to the Centers for Disease Control and Prevention (CDC) classification [35]. For this exploratory study, sarcopenia and sarcopenic obesity were predefined as operational phenotypes rather than clinical diagnoses. Sarcopenia was operationally defined by low muscle strength using the EWGSOP2 sex-specific HGS cut-off of <16 kg for women [41]. Under EWGSOP2, low muscle strength identifies probable sarcopenia, with low muscle quantity or quality required for confirmation. Sarcopenic obesity was operationally defined as the coexistence of BMI-defined obesity and low HGS, recognizing the heterogeneity in definitions and diagnostic approaches used for sarcopenic obesity. Accordingly, participants were classified into four phenotypes: control (BMI < 30 kg/m^2^ and HGS ≥ 16 kg), obesity (BMI ≥ 30 kg/m^2^ and HGS ≥ 16 kg), sarcopenia (BMI < 30 kg/m^2^ and HGS < 16 kg), and sarcopenic obesity (BMI ≥ 30 kg/m^2^ and HGS < 16 kg). The four-group classification and comparisons were prespecified in the study protocol.

#### 2.4.2. Adiposity and Sarcopenia-Related Body-Composition Outcomes

Adiposity outcomes comprised body mass index (BMI), percentage body fat (%BF), fat mass (FM), and fat mass index (FMI). Sarcopenia-related body-composition outcomes comprised fat-free mass (FFM), fat-free mass index (FFMI), and appendicular skeletal muscle index (ASMI). All indices were derived from bioelectrical impedance analysis (BIA), as detailed in Section 2.3.5.

#### 2.4.3. Cardiometabolic Risk Outcomes

Cardiometabolic variables included serum TC, LDL-C, HDL-C and TG concentrations. The TG/HDL-C ratio was calculated as an indirect surrogate of insulin resistance, as previously described [30].

### 2.5. Statistical Analysis

Statistical analyses were conducted using GraphPad Prism version 10.0 (GraphPad Software, Boston, MA, USA). Continuous-variable normality was assessed using the Shapiro–Wilk and Kolmogorov–Smirnov tests. Categorical variables are presented as *n* (%) and compared using the χ^2^ or Fisher’s exact test. Continuous variables are reported as mean ± standard deviation (SD) or median (interquartile range [IQR]) and compared using the unpaired Student’s *t*-test or Mann–Whitney *U* test, respectively. Prespecified pairwise comparisons of circulating irisin between operational phenotypes were performed using the Mann–Whitney U test: sarcopenia versus control, sarcopenic obesity versus control, obesity versus sarcopenia, and sarcopenic obesity versus obesity. These comparisons were selected to examine differences associated with BMI-defined obesity, low handgrip strength, and their coexistence. *p* values were not adjusted for multiple comparisons and were interpreted as nominal within this exploratory analysis.

Spearman’s rank correlation assessed associations between circulating irisin and continuous anthropometric, body-composition, sarcopenia-related, and clinical variables. Exploratory analyses were stratified by menopausal status to characterize associations separately in premenopausal and postmenopausal women. Following the correlation analysis, separate univariable linear regression models were performed for BMI, FMI, and %BF, with circulating irisin as the dependent variable, to quantify the variance in irisin accounted for by each adiposity index. These regression analyses were exploratory and unadjusted. All tests were two-sided, with *p* < 0.05 considered nominally significant. No adjustment for multiple comparisons was applied, and findings were interpreted as exploratory.

## 3. Results

### 3.1. Clinical and Cardiometabolic Characteristics According to Menopausal Status

The study included 79 women, comprising 32 premenopausal and 47 postmenopausal participants. Clinical and cardiometabolic characteristics according to menopausal status are summarized in Table 1. Hypertension was more prevalent among postmenopausal than premenopausal women (55.31% vs. 12.50%; *p* = 0.0006), as was dyslipidemia (82.97% vs. 34.37%; *p* < 0.0001). Postmenopausal women had lower total cholesterol (TC) and low-density lipoprotein cholesterol (LDL-C) concentrations than premenopausal women. Median TC was 4.20 (3.61–5.07) versus 4.90 (4.34–5.55) mmol/L (*p* = 0.0094), and median LDL-C was 2.20 (1.71–2.89) versus 3.10 (2.37–3.42) mmol/L (*p* = 0.0051), respectively.

### 3.2. Obesity and Sarcopenia-Related Indices According to Menopausal Status

As shown in Table 2, obesity and sarcopenia-related indices did not differ significantly between premenopausal and postmenopausal women. BMI, %BF and FMI were comparable between groups, with BMI values of 30.20 ± 6.02 versus 32.00 ± 5.13 kg/m^2^ (*p* = 0.1554), %BF of 37.60 ± 6.35% versus 40.00 ± 4.82% (*p* = 0.0718), and FMI of 12.00 ± 4.34 versus 13.00 ± 3.46 kg/m^2^ (*p* = 0.2790), respectively. Using a BMI cut-off of ≥30 kg/m^2^, obesity was present in 51.89% of the cohort, with no significant difference in prevalence between premenopausal and postmenopausal women (43.75% vs. 57.44%; *p* = 0.2317). When defined by a %BF cut-off of ≥35%, obesity prevalence was 75.94% overall and remained comparable between groups (65.62% vs. 82.97%; *p* = 0.2022).

Sarcopenia-related indices were likewise comparable between menopausal groups. Mean HGS was 17.00 ± 4.20 kg in the overall cohort, whereas median ASM, ASMI and MQI were 18.40 (16.50–20.40) kg, 7.40 (6.95–8.27) kg/m^2^ and 0.90 (0.75–1.08), respectively. None of these measures differed significantly between premenopausal and postmenopausal women (all *p* > 0.05).

### 3.3. Circulating Irisin Levels According to Menopausal Status and Obesity

**Figure 1:**
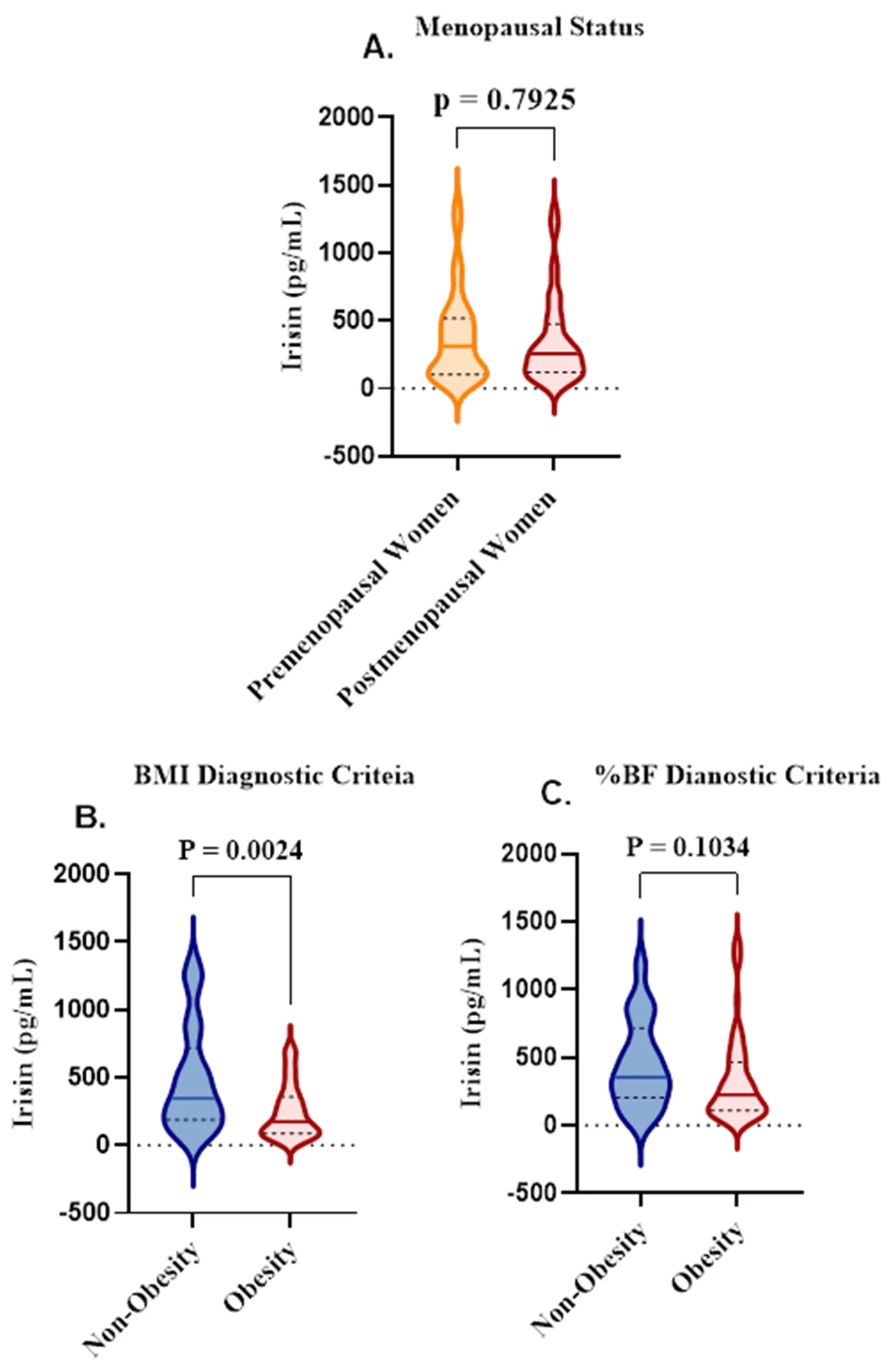

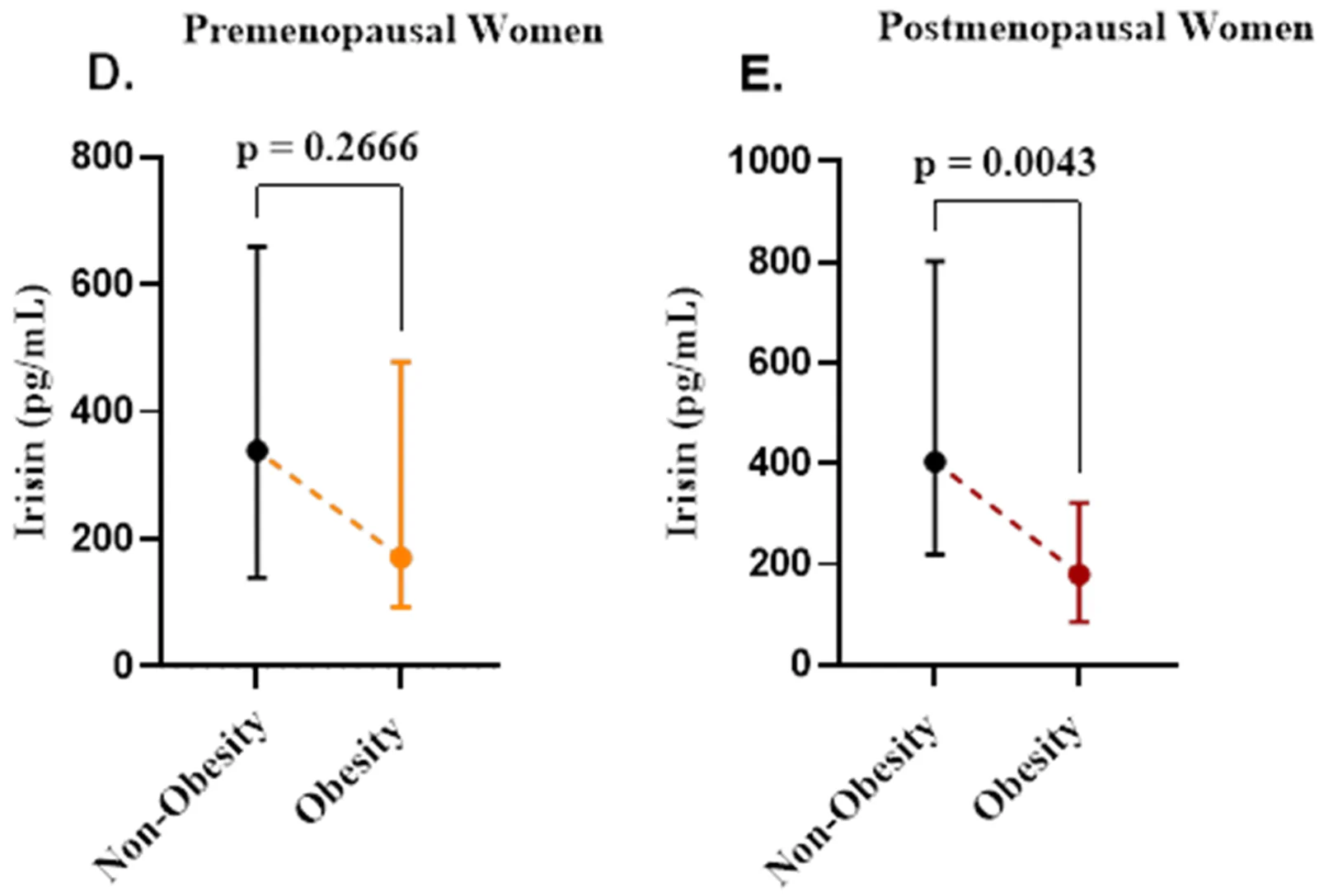
Irisin level according to menopause and obesity. **Notes:** BMI ≥ 30 kg/m^2^ is used as a cut-off to diagnose obesity by body mass index. %BF ≥ 35% is used as a cut-off to diagnose obesity according to body fat percentage. (**D**,**E**) show comparisons based on BMI criteria, with a 30 kg/m^2^ cut-off. The Mann–Whitney test was conducted to compare irisin levels between the two groups. A *p*-value of <0.05 is considered significant. Abbreviations: BMI: body mass index; %BF: percentage body fat.

As shown in Figure 1, circulating irisin concentrations did not differ significantly between premenopausal and postmenopausal women. Median irisin concentration was 264.50 (114.33–509.87) pg/mL in the overall cohort and was comparable between premenopausal and postmenopausal women (256.70 [118.90–476.50] vs. 312.90 [104.00–517.50] pg/mL, respectively; *p* = 0.7925; Figure 1A).

In contrast, BMI-defined obesity was associated with lower circulating irisin concentrations. Using a BMI cut-off of ≥30 kg/m^2^, median irisin concentration was 173.70 (89.03–357.20) pg/mL in women with obesity compared with 347.30 (188.90–716.00) pg/mL in those without obesity (*p* = 0.0024; Figure 1B). When obesity was defined using a %BF cut-off of ≥35%, irisin concentrations were also lower in women with obesity than in those without obesity, although the difference was not statistically significant (225.70 [110.40–466.00] vs. 355.20 [205.30–716.00] pg/mL; *p* = 0.1034; Figure 1C).

Stratification by menopausal status showed that the lower irisin concentrations associated with BMI-defined obesity were observed among postmenopausal women, whereas no statistically significant difference was detected among premenopausal women. In postmenopausal women, median irisin concentration was 179.70 (85.30–321.90) pg/mL in those with obesity versus 403.60 (219.20–801.70) pg/mL in those without obesity (*p* = 0.0043; Figure 1E). Among premenopausal women, corresponding concentrations were 171.10 (92.93–478.10) and 338.70 (138.80–658.90) pg/mL, respectively, with no significant difference (*p* = 0.2666; Figure 1D).

### 3.4. Exploratory Analysis of Circulating Irisin Across Obesity and Sarcopenia Phenotypes

Participants were classified into four operational phenotypic groups: control (n = 23), obesity (n = 22), sarcopenia (n = 14), and sarcopenic obesity (n = 19), with the control group as the reference. Median circulating irisin concentrations were 322.4 (164.10–498.40) pg/mL in controls, 242.1 (102.10–417.20) pg/mL in obesity, 522.8 (196.20–923.30) pg/mL in sarcopenia, and 128.90 (68.57–321.90) pg/mL in sarcopenic obesity.

**Figure 2:**
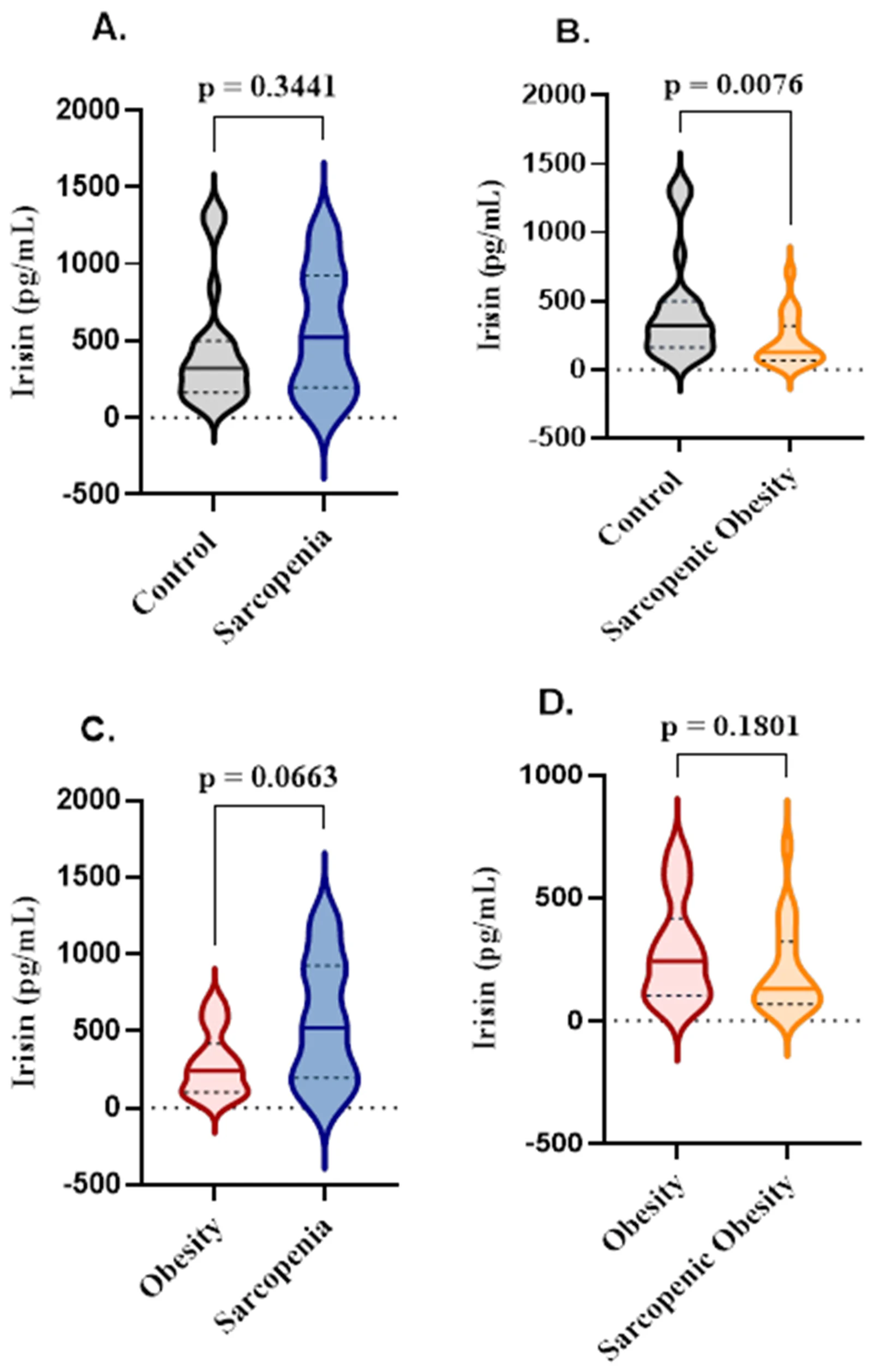
Circulating irisin concentrations across operational sarcopenia and obesity phenotypes. Notes: The groups are defined as follows: control (BMI ˂ 30 kg/m^2^ and HGS ≥ 16 kg), obesity (BMI ≥ 30 kg/m^2^ and HGS ≥ 16 kg), sarcopenia (BMI ˂ 30 kg/m^2^ and HGS ˂ 16 kg), and sarcopenic obesity (BMI ≥ 30 kg/m^2^ and HGS ˂ 16 kg). The Mann–Whitney test was used to compare irisin levels between the two groups. A *p*-value of less than 0.05 indicates statistical significance. Abbreviations: BMI: body mass index; HGS: handgrip strength.

Compared with controls, circulating irisin concentrations were higher in participants with sarcopenia, although the difference was not statistically significant (*p* = 0.3441; Figure 2A). In contrast, concentrations were significantly lower in sarcopenic obesity than in controls (*p* = 0.0076; Figure 2B). Irisin concentrations were also lower in obesity than in sarcopenia, although this difference did not reach statistical significance (*p* = 0.0663; Figure 2C). Similarly, concentrations were lower in sarcopenic obesity than in obesity, but the difference was not statistically significant (*p* = 0.1801; Figure 2D).

### 3.5. Association Between Circulating Irisin and Adiposity Indices

Circulating irisin concentrations were significantly and inversely associated with all three adiposity indices in the total study sample (Table 3). The strongest association was observed with fat mass index (FMI) (r = −0.3835, *p* = 0.0006), followed by body mass index (BMI) (r = −0.3609, *p* = 0.0013) and body fat percentage (%BF) (r = −0.3396, *p* = 0.0025).

### 3.6. Segmental and Whole-Body Composition Across Menopausal Groups

Segmental body composition differed between premenopausal and postmenopausal women, with significantly higher fat percentages in both legs among postmenopausal women (Table 4). Right-leg fat percentage was 45.61 ± 4.43% in postmenopausal women compared with 42.69 ± 5.36% in premenopausal women (*p* = 0.0099), while left-leg fat percentage was 45.61 ± 4.15% versus 42.60 ± 5.17%, respectively (*p* = 0.0062). No significant between-group differences were observed in segmental fat mass or fat percentage of the arms or trunk. Likewise, segmental muscle mass did not differ significantly between menopausal groups across the legs, arms, or trunk.

Whole-body composition showed significant differences in %TBW and the ECW/TBW ratio between menopausal groups (Table 4). %TBW was lower in postmenopausal women than in premenopausal women (42.40 ± 3.37% versus 44.50 ± 4.59%, respectively; *p* = 0.0244), whereas the ECW/TBW ratio was higher in postmenopausal women (47.93 ± 1.43%) than in premenopausal women (46.02 ± 1.91%; *p* < 0.0001). No significant differences were observed in fat mass, fat-free mass (FFM), fat-free mass index (FFMI), total muscle mass, protein mass, bone mass, TBW, ECW, or ICW.

### 3.7. Association Between Circulating Irisin and Sarcopenia-Related Indices

In the total study sample, circulating irisin concentrations were significantly and inversely associated with appendicular skeletal muscle index (ASMI) (r = −0.2773, *p* = 0.0153; Table 5). No significant associations were observed with appendicular skeletal muscle mass (ASM), handgrip strength (HGS), or muscle quality index (MQI).

### 3.8. Association Between Circulating Irisin and Lipid Profile

Circulating irisin concentrations were not significantly associated with any of the assessed lipid parameters, including total cholesterol (TC), HDL-C, LDL-C, triglycerides (TG), or the TG/HDL ratio (Table 5). Although HDL-C showed a positive association with circulating irisin, while TC, LDL-C, TG, and the TG/HDL ratio showed inverse associations, none reached statistical significance.

### 3.9. Univariable Regression Analysis of Circulating Irisin and Adiposity Indices

Separate univariable linear regression models were fitted to examine the associations of circulating irisin with BMI, FMI, and %BF (Table 6). All three adiposity indices showed significant inverse associations with circulating irisin. BMI accounted for the largest proportion of variance in irisin concentrations (β = −0.399, R^2^ = 0.159, F(1,75) = 14.162, *p* = 0.001), followed by FMI (β = −0.390, R^2^ = 0.152, F(1,74) = 13.295, *p* = 0.001) and %BF (β = −0.289, R^2^ = 0.084, F(1,75) = 6.860, *p* = 0.011), accounting for 15.9%, 15.2%, and 8.4% of the variance, respectively.

## 4. Discussion

The principal finding of this study is that circulating irisin was more consistently associated with adiposity than with sarcopenia-related measures in women. Higher BMI, %BF, and FMI were each associated with lower circulating irisin, accounting 15.9%, 8.4%, and 15.2% of its variance, respectively. In contrast, the association with muscle measures was more limited: irisin was inversely associated with ASMI but not with ASM, HGS, or MQI. This pattern suggests that the relationship between irisin and sarcopenia-related indices may differ according to the measure used to characterize muscle status. Notably, irisin concentrations were lowest in women with both obesity and low muscle strength and were significantly lower than those in controls. Together, these findings suggest that adiposity is more consistently related to circulating irisin than muscle strength, while the relationship with muscle status may be more evident when low muscle strength occurs alongside obesity.

The consistent inverse relationship between irisin and adiposity is consistent with the complex biology of this exercise-responsive myokine and its proposed role in muscle–adipose communication [15,16,17,18,19]. Irisin was initially described as a myokine released in response to exercise and implicated in the induction of uncoupling protein 1 and adipose tissue browning [15]. Subsequent research has demonstrated that irisin is also associated with adipose tissue and metabolic regulation, supporting its broader characterization as an adipo-myokine [16,17,18,19,22,23]. Nevertheless, circulating irisin concentrations remain controversial, with substantial heterogeneity across human studies [20,21]. The present findings add to this literature by demonstrating concordant inverse associations across BMI, FMI, and %BF when examined continuously. In contrast, the categorical findings differed according to the obesity criterion: BMI-defined obesity was associated with lower irisin, whereas the corresponding difference using %BF ≥ 35% was not significant. The higher prevalence of obesity defined by %BF than by BMI (75.9% vs. 51.9%) indicates that these criteria characterized adiposity differently within this cohort. The strongest correlation was observed with FMI, an index that directly reflects adipose mass relative to height, further emphasizing the close relationship between circulating irisin and adiposity.

The exploratory univariable regression analyses further quantified these relationships. BMI and FMI accounted for similar proportions of variance in circulating irisin, whereas %BF accounted for a smaller proportion. As these models were unadjusted, they characterize the relationship of each adiposity index with irisin but do not establish independent associations. Nevertheless, the consistent inverse relationships observed across BMI, FMI, and %BF indicate that the association with irisin extends across distinct measures of adiposity rather than being confined to a single metric. Whether lower circulating irisin reflects an adaptive response to excess adiposity, altered muscle–adipose signaling, or broader metabolic remodeling cannot be determined from these cross-sectional data.

The relationship between circulating irisin and muscle-related indices was less consistent. HGS, ASM, and MQI were not significantly associated with irisin, whereas ASMI showed a modest inverse association. The ASMI finding remains exploratory, as its unadjusted relationship with irisin cannot be distinguished from the potential contribution of adiposity. These indices capture different aspects of muscle status: ASMI reflects relative appendicular muscle mass, whereas HGS provides a functional measure of muscle strength and is a primary parameter in sarcopenia assessment [1,4]. This pattern indicates that the relationship between circulating irisin and muscle-related characteristics may differ according to the specific index examined. The observed association with ASMI therefore warrants cautious interpretation and may contribute to understanding the heterogeneous findings previously reported for irisin and muscle-related phenotypes [18,24].

The inverse association between ASMI and irisin nevertheless requires careful interpretation. ASMI is intrinsically related to body size and body composition, while the same cohort demonstrated strong inverse associations between irisin and BMI, FMI, and %BF. Accordingly, the ASMI finding cannot be interpreted as an adiposity-independent sarcopenia signal without adjustment for relevant body-composition measures. The present study therefore supports an association between irisin and muscle-mass phenotype but does not establish irisin as an independent biomarker of sarcopenia. This distinction is particularly important given that reduced muscle strength, rather than muscle mass alone, is central to contemporary sarcopenia definitions [1,4].

The phenotype analysis provides further insight into circulating irisin across operational sarcopenia and obesity phenotypes. Women with sarcopenic obesity had the lowest circulating irisin concentrations and differed significantly from controls, whereas women with sarcopenia without obesity did not show a significant reduction. Indeed, irisin concentrations were numerically higher in the sarcopenia-only group than in controls, although this difference was not statistically significant. Thus, the data do not support a simple inverse gradient between muscle strength and irisin. Rather, the lowest irisin concentrations were observed when obesity and low muscle strength coexisted. Importantly, irisin concentrations did not differ significantly between the sarcopenic-obesity and obesity-only groups, and therefore an additional effect of low muscle strength beyond obesity cannot be inferred. Nevertheless, the observed pattern highlights operational sarcopenic obesity as an informative phenotype for investigating circulating irisin and supports considering adiposity and muscle-strength impairment together when examining its relationship with muscle–adipose characteristics.

Menopausal status provided additional context for the adiposity–irisin relationship. Circulating irisin did not differ significantly between premenopausal and postmenopausal women overall, nor did the groups differ significantly in BMI, FMI, %BF, HGS, ASM, ASMI, or MQI. Stratified analyses, however, revealed an informative pattern: among postmenopausal women, those with obesity had significantly lower circulating irisin than those without obesity, whereas no significant difference was observed among premenopausal women. This pattern highlights the postmenopausal subgroup as a potentially informative context for examining the obesity–irisin relationship. Without a formal menopause-by-obesity interaction test, however, the subgroup finding remains exploratory and does not establish effect modification. These findings provide a focused rationale for further investigation of the obesity–irisin relationship in relation to menopausal status.

The body-composition findings provide additional context for the menopausal-status findings. Although global measures of adiposity and muscle mass did not differ significantly by menopausal status, postmenopausal women had higher fat percentages in both legs. Differences were also observed in total body-water percentage and the extracellular water-to-total body water ratio, whereas overall fat mass, fat-free mass, muscle mass, and related indices were comparable between groups. These findings suggest that menopause in this cohort may have been accompanied more by regional redistribution of adiposity and altered fluid composition than by broad differences in total adiposity or muscle mass. Such changes are consistent with the recognized effects of menopausal transition on body composition and muscle–adipose physiology [25,27,28,42].

The substantial differences in cardiometabolic risk between menopausal groups provide an important additional context. Postmenopausal women had higher prevalence of hypertension and dyslipidemia, as well as significant differences in systolic blood pressure, total cholesterol, and low-density lipoprotein cholesterol. However, circulating irisin was not significantly correlated with individual lipid parameters or the triglyceride-to-high-density lipoprotein ratio. Therefore, within this cohort, the relationship between irisin and metabolic phenotype appears to be more closely aligned with adiposity than with individual circulating lipid measures. These findings also caution against interpreting irisin as a direct surrogate for cardiometabolic risk based solely on lipid profiles.

Several mechanisms may contribute to the observed relationship between adiposity and circulating irisin. Irisin has been proposed to participate in muscle–adipose communication, energy metabolism, and adipose tissue remodeling [15,22,23]. Excess adiposity may alter this signaling environment through changes in adipose tissue function, metabolic demand, inflammation, and substrate handling. Conversely, altered irisin signaling could represent a compensatory response to metabolic stress rather than a simple deficiency. The present cross-sectional data cannot distinguish between these possibilities. Nevertheless, the consistent inverse association across multiple adiposity measures and the particularly low concentrations in women with concurrent obesity and low HGS support the concept that irisin may reflect a broader disturbance in muscle–adipose metabolic communication.

The findings should also be interpreted in light of the heterogeneous human literature on irisin [20,21]. Differences in participant characteristics, sex, age, menopausal status, adiposity, physical activity, muscle phenotype, body-composition methodology, and assay procedures may contribute to the inconsistent associations reported previously [16,17,18,19]. The present study addresses several of these dimensions simultaneously by evaluating women across menopausal groups and examining adiposity alongside multiple measures of muscle quantity, strength, and quality. This integrated approach may explain why the strongest and most reproducible signal in the present cohort was related to adiposity, whereas muscle-related associations were more selective.

This study provides an integrated assessment of circulating irisin in relation to obesity, sarcopenia, sarcopenic obesity, and menopausal status. A key strength is the multidimensional assessment of adiposity and muscle status using BMI, %BF, FMI, segmental body fat, ASM, ASMI, HGS, and MQI. This enabled the relationships of irisin with adiposity, muscle mass, and muscle strength to be examined within the same cohort and allowed obesity and low muscle strength to be examined separately and in combination.

Inclusion of premenopausal and postmenopausal women further enabled exploratory characterization of the obesity–irisin relationship within each menopausal subgroup. Circulating irisin was quantified by ELISA, body composition assessed by BIA, and muscle strength measured by dynamometry using HGS, consistent with the EWGSOP2 emphasis on muscle strength in sarcopenia assessment.

This study was designed as an exploratory investigation of circulating irisin across adiposity and muscle-related characteristics in women. Accordingly, the findings are intended to identify patterns and generate hypotheses rather than establish independent or causal relationships. The cross-sectional design limits causal and temporal inference. The sarcopenia and sarcopenic obesity phenotypes were predefined operationally using HGS, an established indicator of muscle strength. This strength-based classification enabled the examination of circulating irisin across distinct adiposity and muscle-strength profiles, although the findings should be interpreted within these operational definitions when compared with studies incorporating muscle quantity or quality criteria.

The exploratory regression models were unadjusted and were not intended to establish independent associations; therefore, residual confounding by age, menopausal status, medication use, cardiometabolic factors, and unmeasured lifestyle factors cannot be excluded. Whether these associations persist after adjustment for age, menopausal status, and other relevant confounders therefore remains undetermined Subgroup analyses, including those by menopausal status, were exploratory and were not designed to test effect modification, while findings across multiple comparisons require confirmation in larger studies. The modest sample size, particularly within phenotype groups, and recruitment from a single center should be considered when extending these findings to broader populations. Finally, physical activity and other lifestyle factors were not assessed, and a single irisin measurement does not capture temporal variability.

Despite these limitations, the findings identify a consistent pattern: circulating irisin was consistently inversely associated with adiposity, whereas associations with muscle-related indices were less consistent. The lowest irisin concentrations were observed in women with concurrent obesity and low muscle strength. These findings are associative and hypothesis-generating and do not establish irisin as a diagnostic biomarker of sarcopenia or sarcopenic obesity.

The findings provide a basis for a more targeted investigation of irisin in sarcopenic obesity. Longitudinal studies with repeated irisin measurements are needed to determine whether changes in irisin precede or accompany changes in adiposity and muscle status and to clarify the obesity–irisin relationship in relation to menopausal status.

Mechanistic studies should establish whether altered irisin concentrations arise from changes in adiposity, skeletal muscle, or their interaction. This work will be important for determining the biological significance of the low-irisin profile observed in sarcopenic obesity and its potential relevance to the development and characterization of this phenotype.

## 5. Conclusions

Circulating irisin showed consistent inverse associations with adiposity, whereas associations with muscle-related indices were less consistent. The lowest irisin concentrations were observed in women with concurrent obesity and low muscle strength. These findings highlight distinct patterns of circulating irisin across adiposity and muscle-related characteristics in women.

## Figures and Tables

**Table 1:** Clinical characteristics, cardiometabolic risk factors, and lipid profile according to menopausal status.

Parameters	All (n = 79)	Premenopausal Women (n = 32)	Postmenopausal Women (n = 47)	*p* Value
Age (Years)	54.50 (11.37)	43.70 (8.08)	61.90 (6.19)	<0.0001 *
Height (cm)	156.00 (6.03)	156.80 (6.67)	155.50 (5.57)	0.3585 *
Weight (kg)	76.00 (15.08)	75.10 (17.42)	76.70 (13.42)	0.6467 *
HR (bpm)	80.30 (9.55)	81.10 (8.34)	79.70 (10.35)	0.5193 *
SBP (mmHg)	125.50 (14.53)	119.30 (13.32)	129.80 (13.88)	0.0012 *
DBP (mmHg)	71.60 (8.50)	71.00 (8.24)	72.00 (8.73)	0.6032 *
MAP	89.00 (84.00, 97.00)	87.50 (81.00, 93.50)	89.0 (85.00, 98.00)	0.0655 ^
**Cardiometabolic Risk Factors**				
HTN Dx, n (%)				0.0006 **
Yes	30 (37.97)	4 (12.50)	26 (55.31)	
No	47 (59.49)	27 (84.75)	20 (42.55)	
HTN Rx, n (%)				0.0002 **
Yes	28 (36.8)	3 (9.75)	25 (53.19)	
No	48 (63.2)	28 (87.50)	20 (42.55)	
DLD Dx, n (%)				<0.0001 **
Yes	50 (63.29)	11 (34.37)	39 (82.97)	
No	27 (34.17)	20 (62.50)	7 (14.89)	
DLD Rx, n (%)				<0.0001 **
Yes	46 (58.22)	7 (21.87)	39 (82.97)	
No	31 (39.24)	24 (75.00)	7 (14.89)	
**Blood Lipid Profile**				
TC (mmol/L)	4.50 (3.93, 5.26)	4.90 (4.34, 5.55)	4.20 (3.61, 5.07)	**0.0094 ^**
HDL-C (mmol/L)	1.40 (1.18, 1.61)	1.40 (1.25, 1.67)	1.30 (1.17, 1.55)	0.2928 ^
LDL-C (mmol/L)	2.60 (1.93, 3.28)	3.10 (2.37, 3.42)	2.20 (1.71, 2.89)	**0.0051 ^**
TG (mmol/L)	1.10 (0.87, 1.66)	1.00 (0.78, 1.73)	1.10 (0.97, 1.65)	0.1912 ^
TG/HDL-C	0.90 (0.57, 1.26)	0.70 (0.43, 1.24)	0.90 (0.63, 1.31)	0.1207 ^

**Notes:** Continuous variables are expressed as mean and standard deviation (SD) for normally distributed data or median and interquartile range (25, 75%) for non-normally distributed data. *p* < 0.05 indicates statistical significance. Categorical variables are presented as frequencies and percentages. A significant *p*-value is illustrated in bold. The Chi-square and Fisher exact ** tests assessed differences between groups for categorical variables. To compare continuous variables between groups, we used Student’s *t*-test * for normally distributed data and the Mann–Whitney test ^ for data that did not follow a normal distribution. Percentages were calculated using the available observations for each variable; denominators therefore differed across variables. Abbreviations: HR: heart rate; SBP: systolic blood pressure; DBP: diastolic blood pressure; MAP: mean arterial pressure; HTN Dx: hypertension medical diagnosis; HTN Rx: hypertension medical prescription; DLD Dx: dyslipidemia medical diagnosis; DLD Rx: dyslipidemia medical prescription; TC: total cholesterol; HDL-C: high-density lipoprotein cholesterol; LDL-C: low-density lipoprotein cholesterol; TG: triglycerides.

**Table 2:** Obesity and sarcopenia-related indices by menopausal status.

Parameters	All (n = 79)	Premenopausal Women (n = 32)	Postmenopausal Women (n = 47)	*p* Value
**Obesity Indices**				
BMI (kg/m^2^)	31.30 (5.54)	30.20 (6.02)	32.00 (5.13)	0.1554 *
%BF	39.00 (5.57)	37.60 (6.35)	40.00 (4.82)	0.0718 *
FMI (kg/m^2^)	12.60 (3.85)	12.00 (4.34)	13.00 (3.46)	0.2790 *
**Obesity Diagnosis by Different Indices**				
**Obesity by BMI ≥ 30**				0.2317 ^^
Yes	41 (51.89)	14 (43.75)	27 (57.44)	
No	38 (48.10)	18 (56.25)	20 (42.55)	
**Obesity by %BF ≥ 35%**				0.2022 **
Yes	60 (75.94)	21 (65.62)	39 (82.97)	
No	17 (21.51)	10 (31.25)	7 (14.89)	
**Sarcopenia Indices**				
HGS (kg)	17.00 (4.20)	17.10 (4.27)	16.90 (4.20)	0.8919 *
ASM (kg)	18.40 (16.50, 20.40)	18.40 (16.40, 19.40)	18.60 (16.60, 20.40)	0.5572 ^
ASMI (kg/m^2^)	7.40 (6.95, 8.27)	7.30 (6.72, 7.96)	7.50 (6.97, 8.41)	0.3282 ^
MQI	0.90 (0.75, 1.08)	0.91 (0.81, 1.08)	0.90 (0.72, 1.10)	0.7318 ^

**Notes:** Continuous variables are expressed as mean and standard deviation (SD) for normally distributed data or median and interquartile range (25–75%) for non-normally distributed data. *p* < 0.05 indicates statistical significance. Categorical variables are presented as frequencies and percentages. The Chi-square ^^ and Fisher exact ** tests assessed differences between groups for categorical variables. To compare continuous variables between groups, we used Student’s *t*-test * for normally distributed data and the Mann–Whitney test ^ for data that did not follow a normal distribution. Abbreviations: BMI: body mass index; %BF: percentage body fat; FMI: fat mass index; HGS: handgrip strength; ASM: appendicular skeletal muscle; ASMI: appendicular skeletal muscle index; MQI: muscle quality index.

**Table 3:** Association between irisin circulating level and adiposity indices.

Adiposity Index	*r*	*p*
FMI	−0.3835	0.0006 *
BMI	−0.3609	0.0013 *
%BF	−0.3396	0.0025 *

Notes: Spearman’s rank correlation coefficient (r) evaluated the association between irisin and each parameter in the total sample. The correlation is significant at the 0.05 level * (two-tailed). Abbreviations: BMI: body mass index; %BF: percentage body fat; FMI: fat mass index.

**Table 4:** Segmental and whole-body composition by menopausal status.

Parameters	All Women (n = 79)	Premenopausal Women (n = 32)	Postmenopausal Women (n = 47)	*p* Value
**Body Fat Segmental Analysis**				
Fat Mass in Right leg (kg)	6.20 (1.93)	5.90 (2.05)	6.40 (1.84)	0.2769 *
**Fat Percentage in Right Leg (%)**	44.37 (5.01)	42.69 (5.36)	45.61 (4.43)	0.0099 *
Fat Mass in Left Leg (kg)	5.57 (4.90, 7.50)	5.10 (4.30, 7.30)	6.19 (5.00, 7.80)	0.0845 ^
**Fat Percentage in Left Leg (%)**	44.42 (4.79)	42.60 (5.17)	45.61 (4.15)	0.0062 *
Fat Mass in Right Arm (kg)	1.58 (1.30, 2.40)	1.50 (1.20, 2.30)	1.80 (1.40, 2.50)	0.1569 ^
Fat Percentage in Right Arm (%)	44.07 (7.65)	43.12 (8.32)	44.80 (7.17)	0.3344 *
Fat Mass in Left Arm (kg)	1.70 (1.30, 2.50)	1.49 (1.20, 2.20)	1.90 (1.40, 2.70)	0.0603 ^
Fat Percentage in Left Arm (%)	44.07 (7.65)	43.11 (8.32)	44.82 (7.17)	0.3344 *
Fat Mass in Trunk (kg)	14.21 (4.75)	13.20 (5.08)	14.90 (4.47)	0.1326 *
Fat Percentage in Trunk (%)	35.70 (30.00, 39.00)	33.30 (30.00, 39.60)	36.28 (31.70, 38.60)	0.4050 ^
**Body Muscle Segmental Analysis**				
Muscle Mass in Right leg (kg)	7.11 (6.40, 7.80)	7.07 (6.50, 7.70)	7.12 (6.40, 7.90)	0.9172 ^
Muscle Mass in Left Leg (kg)	7.02 (6.40, 7.80)	7.01 (6.40, 7.50)	7.11 (6.30, 7.80)	0.7590 ^
Muscle Mass in Right Arm (kg)	2.08 (0.31)	2.03 (0.36)	2.10 (0.26)	0.1806 *
Muscle Mass in Left Arm (kg)	2.20 (0.35)	2.13 (0.42)	2.24 (0.30)	0.1935 *
Muscle Mass in Trunk (kg)	24.60 (22.50, 27.20)	24.30 (22.00, 27.50)	24.58 (23.10, 27.20)	0.3972 ^
**Body Composition Analysis**				
Fat Mass (kg)	30.77 (9.68)	29.71 (11.33)	31.62 (8.43)	0.3991 *
Fat Free Mass (kg)	45.20 (6.70)	44.31 (8.47)	45.83 (5.20)	0.3911 *
FFMI	18.60 (2.70)	18.11 (3.26)	18.90 (2.21)	0.2353 *
Muscle Mass (kg)	42.40 (39.00, 47.60)	41.51 (37.90, 47.50)	43.20 (39.80, 48.40)	0.3634 ^
Protein (kg)	11.00 (1.40)	10.80 (1.64)	11.20 (1.21)	0.2628 *
Bone Mass (kg)	2.30 (2.10, 2.50)	2.30 (2.10, 2.60)	2.30 (2.10, 2.50)	0.7856 ^
TBW (kg)	32.60 (4.15)	32.60 (4.80)	32.60 (3.71)	0.9887 *
% TBW	43.30 (4.01)	44.50 (4.59)	42.40 (3.37)	0.0244 *
ECW (kg)	15.00 (13.70, 17.10)	14.70 (13.00, 16.80)	15.10 (14.10, 17.30)	0.1751 ^
ICW (kg)	17.20 (2.03)	17.49 (2.20)	17.01 (1.89)	0.2524 *
ECW/TBW (%)	47.11 (1.87)	46.02 (1.91)	47.93 (1.43)	<0.0001 *

Notes: Continuous variables are represented as mean and standard deviation (SD) for normally distributed data or median and interquartile range (25, 75%) for non-normally distributed data. *p* < 0.05 indicates statistical significance. A significant *p*-value is illustrated in bold. The Chi-Square To compare continuous variables between groups, we used the Student’s t-test * for normally distributed data and the Mann–Whitney test ^ for data that did not follow a normal distribution. Abbreviations: FFMI: fat-free mass index; TBW: total body water; % percentage of total body water; ECW: extracellular water; ICW: intracellular water.

**Table 5:** Associations of circulating irisin with sarcopenia-related indices and lipid profile.

	Parameter	Correlation Coefficient (r)	*p* Value
**Sarcopenia Indices**	HGS (kg)	0.08645	0.4547
	ASM (kg)	−0.1608	0.1625
	**ASMI (kg/m^2^)**	**−0.2773**	0.0153 *
	MQI	0.1534	0.1829
**Blood Lipid Profile**	TC (mmol/L)	−0.03411	0.7669
	HDL-C (mmol/L)	0.1680	0.1414
	LDL-C (mmol/L)	−0.1097	0.3388
	TG (mmol/L)	−0.1060	0.3555
	TG/HDL	−0.1460	0.2022

Notes: Spearman’s rank correlation coefficient (r) evaluated the strength and direction of the association between the irisin circulating level and each parameter in the total sample. The correlation is significant at the 0.05 level * (two-tailed). Significant *p*-value indicated in bold. Abbreviations: HGS: handgrip strength; ASM: appendicular skeletal muscle mass; ASMI: appendicular skeletal muscle index; MQI: muscle quality index; TC: total cholesterol; HDL-C: high-density lipoprotein cholesterol; LDL-C: low-density lipoprotein cholesterol; TG: triglycerides.

**Table 6:** Univariable linear regression of circulating irisin on adiposity indices.

Adiposity Index	β	*R* ^2^	*F*	*t*	*p*
%BF	−0.289	0.084	6.860	−2.619	0.011
BMI	−0.399	0.159	14.162	−3.763	0.001
FMI	−0.390	0.152	13.295	−3.643	0.001

Notes: Separate simple linear regression models were fitted for each adiposity index, with circulating irisin concentration as the dependent variable. β, standardized regression coefficient; R^2^, coefficient of determination. Statistical significance was defined as *p* < 0.05. Models were exploratory and unadjusted.

## Data Availability

The data presented in this study are available upon reasonable request from the corresponding author.

## Supplementary Materials

The following supporting information can be downloaded at: https://www.mdpi.com/article/10.3390/nu18213531/s1, Supplementary Figure S1. Flow diagram of participant recruitment, eligibility assessment, and inclusion in serum irisin analyses.

## Abbreviations

The following abbreviations are used in this manuscript:

**%BF**	percentage body fat
**ASM**	appendicular skeletal muscle mass
**ASMI**	appendicular skeletal muscle index
**BMI**	body mass index
**FFMI**	fat-free mass index
**FMI**	fat mass index
**HGS**	handgrip strength
**MQI**	muscle quality index
