# Supplementary material for: Association of Circulating Irisin with Adiposity, Sarcopenia-Related Indices, and Phenotypes in Premenopausal and Postmenopausal Women
Source: Nutrients. 2026 Oct 27;18(21):3531. doi: 10.3390/nu18213531 (PMC13765228; doi:10.3390/nu18213531)

Supplementary

Supplementary Figure S1. Flow diagram of participant recruitment, eligibility assessment, and inclusion in serum irisin analyses.

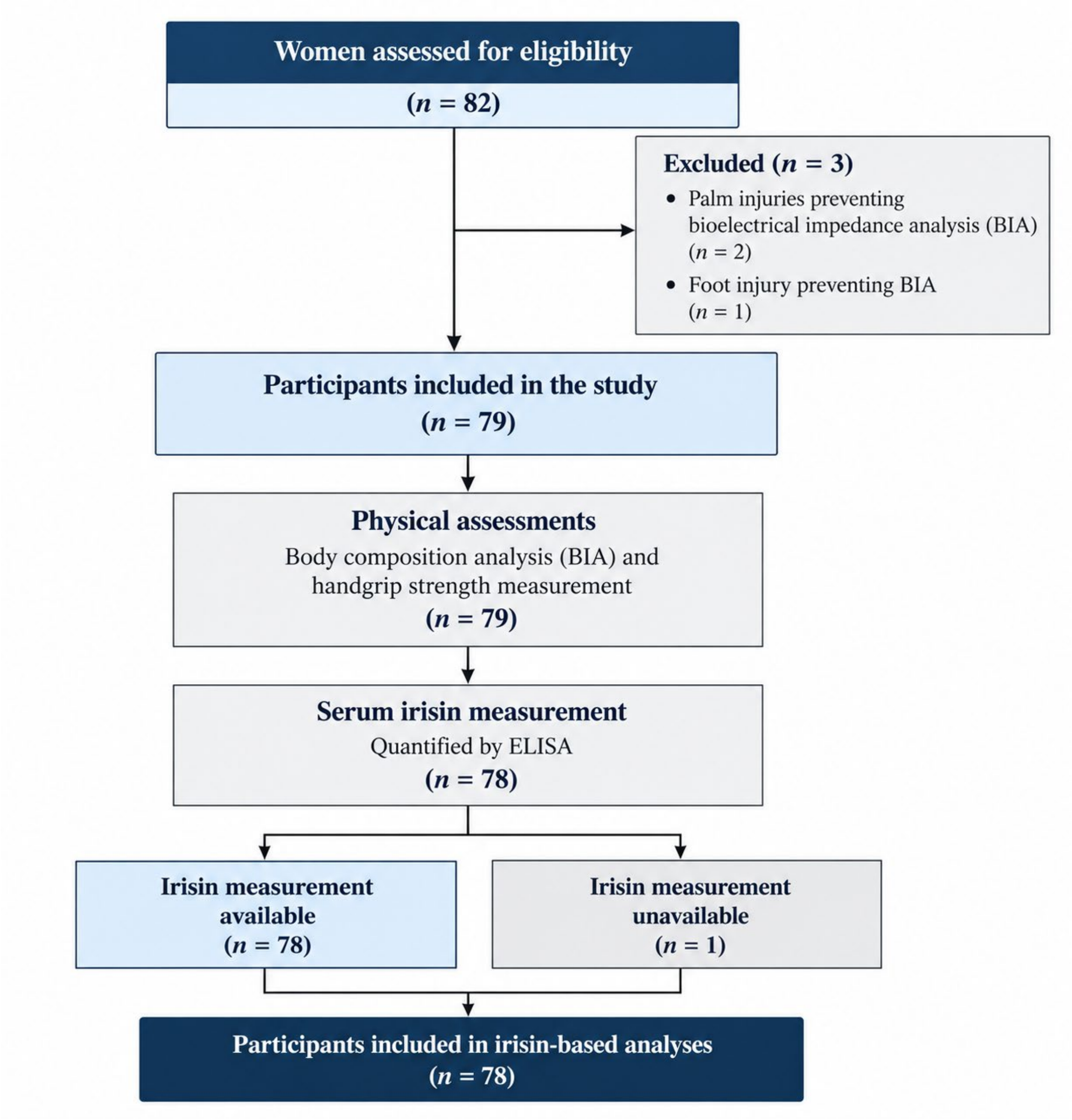

Supplement: Supplementary file 1 [file nutrients-18-03531-s001.zip › nutrients-4559336-supplementary.pdf]
